# DYADIC HEALTH SCIENCE IN GERONTOLOGY: RECENT ADVANCES AND INTERDISCIPLINARY PERSPECTIVES

**DOI:** 10.1093/geroni/igad104.1590

**Published:** 2023-12-21

**Authors:** Lynn Martire, Karen Lyons, Christine Proulx

## Abstract

Close others impact health in profound ways, and many of the critical pathways can only be understood by examining the dynamics that emerge between two people. This symposium will present new theoretical and methodological advances in dyadic health science, and invite discussion of differing perspectives on the scope of dyadic research. Dr. Wilson will overview the essential components of rigorous dyadic health research and explain the range of approaches used to define dyadic research, setting the stage for the cutting-edge work described in the subsequent presentations. Drs. Novak and Yorgason will discuss the importance of matching research questions with dyadic data and how to best model congruence/similarity, difference scores, accuracy or bias, and idealization between partners. Dr. Pauly will describe a recently proposed developmental-contextual model of couple synchrony, and present findings from research using a smartwatch and smartphone system (DyMand) that captures dyadic interactions. Dr. Polenick will present findings from research examining effects of incongruent and discordant health conditions within older couples on depressive symptoms. Dr. Christine Proulx, an internationally recognized scholar of close relationships in late life, will offer perspective on these advances and critical future directions for the field. This is a Dyadic Research on Health and Illness Across the Adult Lifespan Interest Group Sponsored Symposium.

